# Dynamics of Myelin-Specific T-Cell Repertoires Mirror Disease Activity and Central Nervous System Trafficking in Multiple Sclerosis

**DOI:** 10.3390/ijms27157034

**Published:** 2026-08-05

**Authors:** Gabriele Di Sante, Assunta Bianco, Matteo Lucchini, Anna Maria Stabile, Alessandra Pistilli, Marco Bisurgi, Alessandra Cicia, Desirée Bartolini, Loredana Ingrosso, Mario Rende, Maria Concetta Geloso, Simona Rolla, Francesco Ria, Massimiliano Mirabella

**Affiliations:** 1 Department of Medicine and Surgery, Section of Human, Clinical and Forensic Anatomy, University of Perugia, 06123 Perugia, Italy; gabriele.disante@unipg.it (G.D.S.); anna.stabile@unipg.it (A.M.S.); alessandra.pistilli@unipg.it (A.P.); mario.rende@unipg.it (M.R.); 2 Centro di Ricerca Per la Sclerosi Multipla “Anna Paola Batocchi”, Department of Neuroscience, Università Cattolica del Sacro Cuore, 00168 Rome, Italy or assunta.bianco@policlinicogemelli.it (A.B.); or matteo.lucchini@policlinicogemelli.it (M.L.); marco.bisurgi01@icatt.it (M.B.); alessandra.cicia@unicatt.it (A.C.); or massimiliano.mirabella@policlinicogemelli.it (M.M.); 3 Multiple Sclerosis Unit, Fondazione Policlinico Universitario A. Gemelli IRCCS, 00168 Rome, Italy; 4 Department of Pharmaceutical Sciences, University of Perugia, 06123 Perugia, Italy; desiree.bartolini@unipg.it; 5 Department of Infectious Diseases, Istituto Superiore di Sanità, 00161 Rome, Italy; loredana.ingrosso@iss.it; 6 European Program for Public Health Microbiology Training (EUPHEM), European Centre for Disease Prevention and Control (ECDC), 171 83 Stockholm, Sweden; 7 Department of Neuroscience, Section of Human Anatomy, Università Cattolica del Sacro Cuore, 00168 Rome, Italy; 8 Gemelli Science and Technology Park (GSTeP)-Organoids Research Core Facility, Fondazione Policlinico Agostino Gemelli IRCCS, 00168 Rome, Italy; 9 Department of Clinical and Biological Sciences, University of Torino, 10043 Orbassano, Italy; simona.rolla@unito.it; 10 Department of Translational Medicine and Surgery, Section of General Pathology, Università Cattolica del Sacro Cuore, 00168 Rome, Italy; francesco.ria@unicatt.it

**Keywords:** T-cell receptor repertoire, antigen-specific immunity, immune biomarkers, neuroinflammation, immunogenetics

## Abstract

Autoreactive lymphocytes are thought to contribute to tissue injury in the pathogenesis of multiple sclerosis (MS). However, reliable biomarkers reflecting myelin-specific immune responses and disease activity remain limited. In HLA-DRB1*15:01-positive individuals, the myelin basic protein (MBP) epitope MBP85–99 represents a well-characterized immunodominant target of CD4+ T cells. Using classical spectratyping, we characterized the peripheral T-cell receptor (TCR) β-chain repertoire reactive to MBP85–99 in individuals with MS, according to their HLA-DRB1 haplotypes. Selected rearrangements were further evaluated in cerebrospinal fluid (CSF), cytokine-defined T-cell subsets, and longitudinal samples collected during interferon beta-1a treatment. A restricted set of shared (“public”) MBP85–99-reactive TCR rearrangements was identified in individuals with MS and differed from the repertoire observed in healthy controls. These TCRs were more frequently detected during periods of active disease than during remission. Two disease-associated public rearrangements were enriched in active MS, whereas one rearrangement was preferentially observed in healthy controls. HLA-DRB1*15:01-restricted MBP85–99-specific public TCR signatures were associated with inflammatory disease activity in MS and may help distinguish pathogenic from non-pathogenic autoreactive responses. In a longitudinal sub-cohort (n = 15, ~10-year follow-up), the RSI of the principal disease-associated rearrangement TRBV19–TRBJ2.4 correlated positively with cumulative relapse burden (Spearman ρ = 0.782, *p* = 0.002), providing preliminary evidence that these TCR signatures reflect long-term inflammatory disease activity. These findings support further validation of antigen-specific TCR profiling as a biomarker strategy in MS using contemporary high-throughput immune repertoire technologies.

## 1. Introduction

Multiple sclerosis (MS) is a chronic immune-mediated disease of the central nervous system (CNS) characterized by inflammation, demyelination, axonal injury, and progressive neurological disability. Although its pathogenesis is multifactorial, adaptive immune responses involving CNS-reactive T and B lymphocytes are considered key contributors to disease initiation and perpetuation [1,2,3,4]. Recent single-cell studies have further refined this view by identifying disease- and compartment-specific immune signatures in blood and cerebrospinal fluid (CSF) of patients with MS [5,6,7].

Genetic susceptibility to MS is strongly influenced by the major histocompatibility complex (MHC), with HLA-DRB1*15:01 representing the most consistently associated risk allele across populations [8,9,10,11]. Recent genetic and repertoire-based studies have further supported the concept that HLA risk variants shape the composition and characteristics of the T-cell receptor (TCR) repertoire in MS [12].

Despite major therapeutic advances, reliable biomarkers reflecting disease-relevant immune activity and predicting response to disease-modifying therapies remain limited. Clinical relapses and magnetic resonance imaging (MRI) activity remain central to monitoring, but they provide only indirect and often retrospective information on underlying immunological activity. Blood and CSF biomarkers such as neurofilament light chain are increasingly relevant for assessing neuroaxonal injury and prognosis, but they do not directly identify antigen-specific autoimmune responses [13,14,15].

Among candidate autoantigens implicated in MS, myelin basic protein (MBP) has been extensively studied. In HLA-DRB115:01-positive individuals, the MBP85–99 peptide is a well-characterized immunodominant epitope recognized by CD4+ T cells. Structural studies demonstrated the binding of MBP85–99 to HLA-DR2/DRB115:01, and MBP85–99/HLA-DR2 complexes have been visualized in MS lesions [16,17,18,19].

However, MBP-reactive T cells can also be detected in healthy individuals carrying the same HLA background [20,21], indicating that the mere presence of autoreactivity is insufficient to explain the disease. Therefore, qualitative features of the response, including clonotypic composition, activation state, cytokine profile and CNS trafficking, should be more informative than antigen recognition alone.

TCR repertoire analysis provides a powerful strategy for dissecting antigen-specific immune responses at clonal resolution. Shared, or “public”, TCR rearrangements that recur across individuals may identify biologically meaningful immune signatures. Spectratyping (immunoscope) analysis is a robust and sensitive approach to detect antigen-driven T-cell expansions and has been successfully applied to the study of autoimmune and inflammatory settings [22,23,24,25].

Mirroring these observations in humans, naïve Swiss Jim Lambert (SJL/J) mice display a spontaneous response to the myelin epitope PLP139-151, but they do not develop experimental autoimmune encephalomyelitis (EAE) unless challenged with a peptide encompassing this same epitope in adjuvant [26]. Following the challenge, the pre-immune repertoire is recruited to the draining lymph nodes, where T-cell priming of the immune repertoire occurs, while the former is depleted and never reappears [27,28]. Through the spectratyping technique it was also possible to detect and follow the encephalitogenic T cells within the CNS in the murine model of MS [29]. Furthermore, in rheumatoid arthritis (RA) and in other immune-mediated disorders, TCR repertoires are associated with disease activity and response to therapy [24,30] and are associated with resistance to methotrexate therapy in RA [23].

In the present study, we investigated the MBP85–99-reactive TCRβ repertoire in HLA-DRB1*15:01-positive persons with MS (PwMS) during active relapsing or remitting phases or with a first demyelinating event and compared it with HLA-matched healthy donors. We aimed to determine whether shared clonotypic signatures are associated with disease activity, CSF compartmentalization, and modulation during interferon beta-1a treatment.

## 2. Results

### 2.1. Identification of the TCR-Beta Repertoire Specific for MBP85–99 in HLA-DRB1*15:01^+^ MS Patients

The use of the immunoscope analysis allowed the spectratyping of the repertoires of the beta chain of TCR [31] to investigate T-cell-specific rearrangements for the immunodominant epitope MBP85–99 and the less immunogenic MBP111–129 in HLA-DRB1*15 + PwMS. The full cohort of 37 HLA-DRB1*15:01-positive subjects was composed of seven healthy donors (HDs), eight with a first demyelinating event (FDE), 11 with active relapsing disease (REL), and 11 in remission (REM).

Immunoscope/spectratyping analysis was applied to a pool of cDNA samples from PBMCs of seven HLA-DRB1*15:01-positive PwMS, cultured in the absence or pre-sence of MBP85–99. This pooling strategy was used for initial repertoire discovery, followed by individual-level validation.

**Figure 1:**
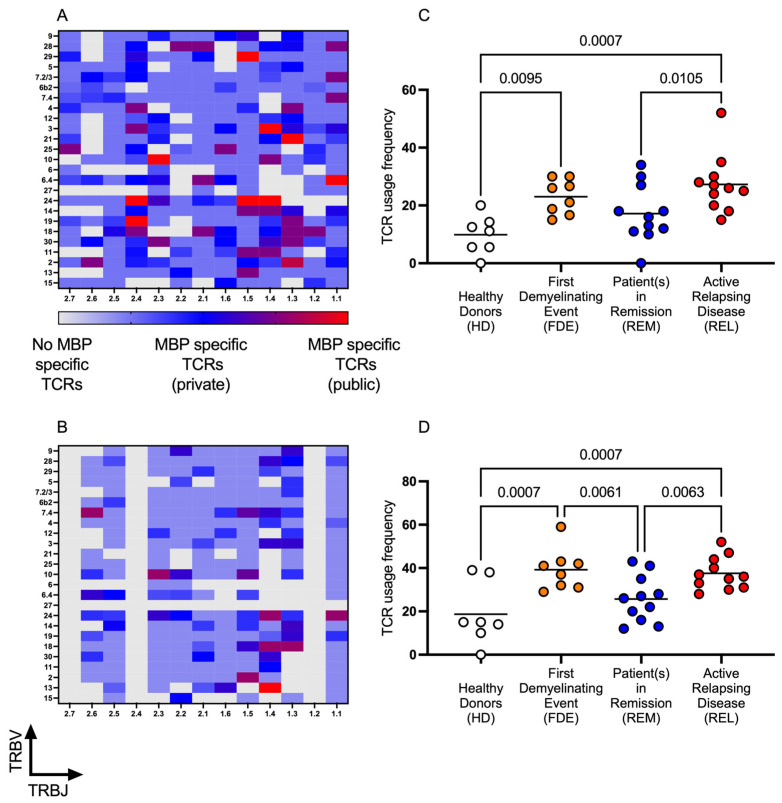
(**A**) Candidate MBP85–99-specific TRBV-TRBJ rearrangements identified by pooled immunoscope analysis. MBP85–99-specific expansions shown in blue; rearrangements shared across patients in red. (**B**) MBP111–129-specific repertoire identified in the same analysis. (**C**) Frequency of MBP85–99-specific TCR usage per individual across groups (healthy donors [HD], first demyelinating event [FDE], active relapsing disease [REL], remission [REM]); (**D**) Frequency of MBP111–129-specific TCR usage per individual across groups. For B and D, a one-way ANOVA test was used, controlling the false discovery (correction with the two-stage step-up method of Benjamini, Krieger and Yecutieli).

Twenty candidate MBP85–99-specific TRBV-TRBJ rearrangements were identified in the stimulated pool compared with unstimulated controls (Figure 1A). Individual validation in each patient confirmed that eight of these twenty rearrangements were shared among PwMS, with two being exclusive to patients with established MS (not detected in the first demyelinating event). Three rearrangements (TRBV28-TRBJ2.2, TRBV3-TRBJ2.4, and TRBV24-TRBJ1.5) were also expanded upon stimulation with the subdominant epitope MBP111–129, while MBP111–129 stimulation additionally expanded a non-overlapping, epitope-specific repertoire (Figure 1B), with different specificity/clonality. These 20 candidate rearrangements were subsequently tested across the full cohort of 37 HLA-DRB1*15:01-positive subjects (individual-level RSI values for all 20 rearrangements across the full cohort are shown in Figure S1).

In the subgroups of PwMS, the first demyelinating event and active relapsing disease showed a significantly higher frequency of MBP85–99-specific TCR rearrangements compared to healthy donors. No significant difference was observed between remission and healthy donors (Figure 1C). Notably, first demyelinating event subjects displayed a significantly broader MBP85–99-specific repertoire than active relapsing disease patients, suggesting dynamic repertoire remodeling during disease progression, consistent with active clonal selection following disease onset. The difference between remitting and active relapsing disease was barely statistically significant (*p* = 0.01), possibly reflecting limited statistical power in this subgroup comparison and potentially because in the remitting group there was a heterogeneous distribution of patients.

For the MBP111–129-specific repertoire, a small but detectable response was observed in HD, consistent with non-pathogenic autoreactivity to this subdominant epitope. FDE patients showed a significantly enlarged MBP111–129-specific repertoire compared to HD, while REM patients showed a reduction with respect to the first demyelinating event. Active relapsing disease (REL) remained significantly elevated compared with both HD and REM (q = 0.0007 and q = 0.0063, respectively) and did not differ significantly from FDE (q = 0.25), indicating that, unlike the pattern observed for MBP85–99, expansion of the MBP111–129-specific repertoire persists into the active relapsing phase rather than being confined to disease onset (Figure 1D). This pattern is reminiscent of epitope spreading described in experimental models of autoimmunity [32,33].

### 2.2. Disease/Health Associated Public MBP85–99-Specific TCRβ Rearrangements

**Figure 2:**
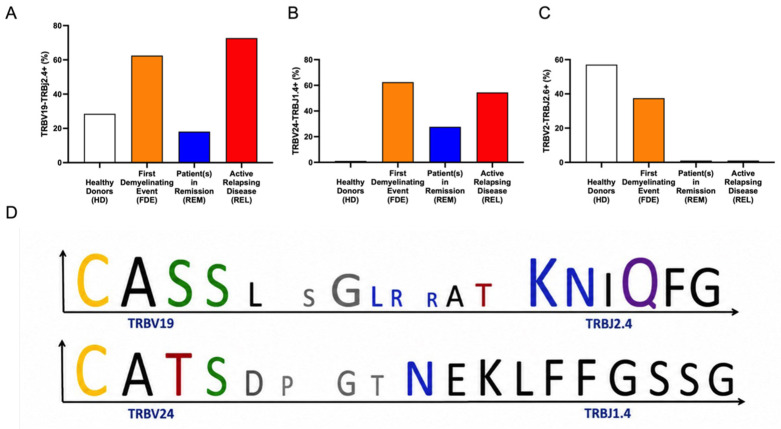
Frequency of expansion (%) of TRBV19-TRBJ2.4 108bp (**A**), TRBV24-TRBJ1.4 125bp (**B**), and TRBV2-TRBJ2.6 (**C**) in healthy donors (HD; n = 7), those with a first demyelinating event (FDE; n = 8), those in remission (REM; n = 11), and those with active relapsing disease (REL; n = 11). (**D**) CDR3 amino acid sequences of TRBV19-TRBJ2.4 and TRBV24-TRBJ1.4 from multiple active-phase patients. Gray shading indicates amino acid residues conserved across patients, highlighting the recurrent motif shared within each rearrangement; unshaded residues indicate variable positions (see also Tables S1 and S2).

Among the validated rearrangements, three exhibited a ‘public’ pattern, i.e., they were shared across multiple unrelated HLA-DRB1*15:01-positive subjects and showed differential association with disease status (Figure 2).

The TRBV19-TRBJ2.4 rearrangement (108 bp) was expanded in 5/8 persons with a first demyelinating event, 8/11 active relapsing disease PwMS, 2/11 remitting PwMS, and 2/7 healthy donors, establishing it as a disease-associated public rearrangement enriched in active phases of MS (Figure 2A). The TRBV24-TRBJ1.4 rearrangement (125 bp) was detected in 5/8 of the group with a first demyelinating event, 6/11 of the active relapsing disease PwMS, and 3/11 of the remitting PwMS, representing a semi-public rearrangement present in approximately 50% of subjects across disease states (Figure 2B). These findings indicate the presence of distinct, convergent rearrangements preferentially associated with active MS.

To confirm antigen-driven selection of these rearrangements, the CDR3 regions of TRBV19-TRBJ2.4 and TRBV24-TRBJ1.4 were sequenced in multiple active-phase patients. Recurrent amino acid motifs were identified in the CDR3 of both rearrangements across several HLA-DRB1*15:01-positive individuals with active MS (Figure 2D, Tables S1 and S2), consistent with convergent antigen-driven selection of these rearrangements [34,35].

Analogous to what has been described in the SJL/J murine model [26], we also identified a public MBP85–99-specific TCR rearrangement enriched in healthy individuals. The TRBV2-TRBJ2.6 rearrangement was detected in 4/7 HD and 3/8 FDE subjects but was absent in all patients with established MS diagnoses, both REL and REM. Conversion to clinically definite MS was ascertained at a standardized 6-month clinical reassessment (the timepoint of the second blood draw), applied uniformly to all eight FDE participants. Within this window, five of the eight FDE patients converted to clinically definite MS and three did not; none of the five FDE patients who converted to clinically definite MS within follow-up carried this rearrangement, while three non-converters did (Figure 2C). Representative immunoscope profiles illustrating these public rearrangements across disease groups are shown in Supplementary Figure S3.

In comparison, the frequency of the individual-level expansion (RSI ≥ 2) of the 20 candidate TCRs, described in Figure S1, was displayed (n/N tested per group) in Table S3.

Taken together, these data identify three public MBP85–99-specific TCRβ rearrangements in HLA-DRB1*15:01-positive subjects: two associated with active MS and one potentially associated with a non-pathogenic autoreactive state in health, which may also correlate with a slower rate of FDE-to-MS conversion.

### 2.3. Disease-Associated TCRβ Rearrangements Are Enriched in the CSF During Active MS

To evaluate whether T cells carrying disease-associated public TCRs traffic to the CNS during active disease, we analyzed MBP85–99-specific rearrangements in mononuclear cells of CSF from 44 patients (first demyelinating event, active relapsing disease, and remission) without prior in vitro stimulation. Sixteen samples were from HLA-DRB1*15:01-positive patients (details in Table S4).

**Figure 3:**
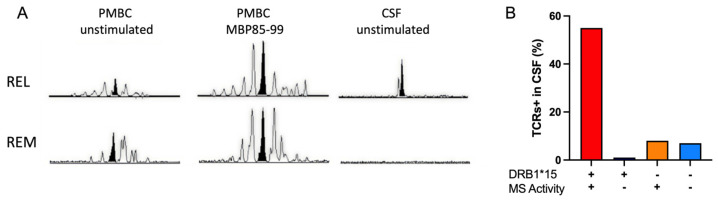
(**A**) Representative immunoscope profiles of TRBV19-TRBJ2.4 in PBMC and CSF from a PwMS with active relapsing disease and a PwMS in remission. RSI = Rate Stimulation Index; dotted line at RSI = 2 indicates threshold for expansion. (**B**) Frequency of detection of TRBV19–TRBJ2.4 (108 bp) and TRBV24–TRBJ1.4 (124–5 bp) in CSF from 44 patients stratified by HLA-DRB1*15:01 status and disease activity (first demyelinating event [FDE]+ active relapsing disease [REL] vs. remission [REM]). Data detailed in Table S4. Fisher’s exact test, DRB1*15:01+ active MS vs. DRB1*15:01+ remission, *p* = 0.034; DRB1*15:01-negative acute, *p* = 0.023; DRB1*15:01-negative remission, *p* = 0.015.

The TRBV19-TRBJ2.4 (108 bp) and TRBV24-TRBJ1.4 (124-5 bp) rearrangements were detectable in 5/9 (56%) CSF samples from HLA-DRB1*15:01-positive patients in the active disease phase (first demyelinating event or active relapsing disease). In contrast, neither rearrangement was detected in any of the seven HLA-DRB1*15:01-positive patients in remission (*p* = 0.034, Fisher’s exact test). As a specificity control, these rearrangements were detected in only 2/28 CSF samples from HLA-DRB1*15:01-negative patients (1/13 in acute presentation, *p* = 0.023; 1/15 in remission; *p* < 0.015; Fisher’s exact test). Representative immunoscope profiles from a PwMS with active relapsing disease and a PwMS in remission are shown in Figure 3A, demonstrating the antigen-specific CSF enrichment in active disease only. These findings confirm HLA-restricted, disease-activity-dependent CNS trafficking of MBP85–99-specific T cells (Figure 3B) [36,37].

### 2.4. Longitudinal Changes in the MBP85–99-Specific TCRβ Repertoire During Interferon Beta-1a Treatment

To assess whether the MBP85–99-specific TCR repertoire reflects the immunological effects of treatment, we analyzed serial PBMC samples from five HLA-DRB1*15:01-positive MS patients at baseline (active disease, prior to DMT) and after 6 months of interferon beta-1a (IFN-β) therapy (during clinical and radiological remission). Individual-level RSI profiles across all rearrangements at the three timepoints are shown in Supplementary Figure S2.

**Figure 4:**
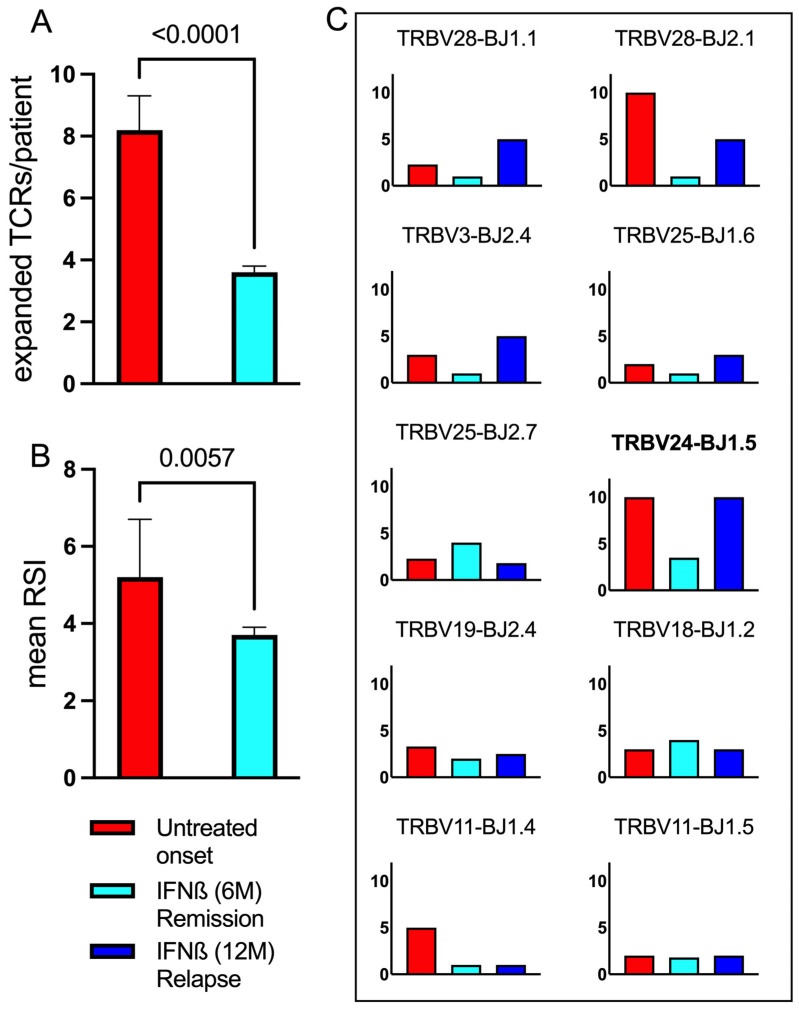
(**A**) Mean number of expanded MBP85–99-specific TCR clones per PwMS at baseline (active MS) and after 6 months of IFN-β therapy. (**B**) Mean RSI of expanded rearrangements at the same two time points. (**C**) Longitudinal TCRβ rearrangement profile in one PwMS at baseline (red bars, active MS without therapy), 6 months (light blue bars, remission under IFN-β), and 12 months (blue bars, relapse under IFN-β). Data shown as RSI per rearrangement; dotted line at RSI = 2 indicates expansion threshold. See Supplementary Figure S2 for the complete rearrangement-level RSI profile of this patient at all three timepoints.

In this exploratory longitudinal subset, IFN-β treatment coincided with a significant reduction in both the breadth (number of expanded MBP85–99-specific rearrangements) and intensity (mean RSI) of the antigen-specific repertoire (Figure 4A,B). In a detailed longitudinal case examined at three time points (baseline, 6-month remission, 12-month relapse), 5/10 disease-associated rearrangements were undetectable during remission; at the time of relapse, the repertoire largely recapitulated the baseline pattern (Figure 4C). One rearrangement (TRBV11-TRBJ1.4) appeared stably deleted after IFN-β treatment. These exploratory findings, while limited by small sample size, are consistent with the known immunomodulatory effects of IFN-β on T-cell activation, cytokine signaling, and leukocyte trafficking [38,39,40].

### 2.5. Longitudinal Clinical Correlates of Disease-Associated TCRβ Rearrangements

To explore associations between the identified MBP85–99-specific TCR repertoire and long-term clinical disease characteristics, we performed a retrospective cross-sectional analysis integrating individual-level RSI data from the primary spectratyping cohort with available clinical parameters retrieved from the institutional database at approximately 10-year follow-up. This analysis was restricted to the 15 subjects for whom both TCR spectratyping data and complete follow-up records were available (six with first demyelinating events, four with active relapsing disease, five in remission at the time of sampling; Table 1 and Table S5). Spearman rank correlations were computed between RSI values for six key rearrangements (TRBV19-TRBJ2.4, TRBV24-TRBJ1.4, TRBV2-TRBJ2.6, TRBV28-TRBJ2.1, TRBV3-TRBJ2.4, TRBV24-TRBJ1.5) and continuous clinical variables including EDSS, number of relapses, and age at sampling.

**Figure 5:**
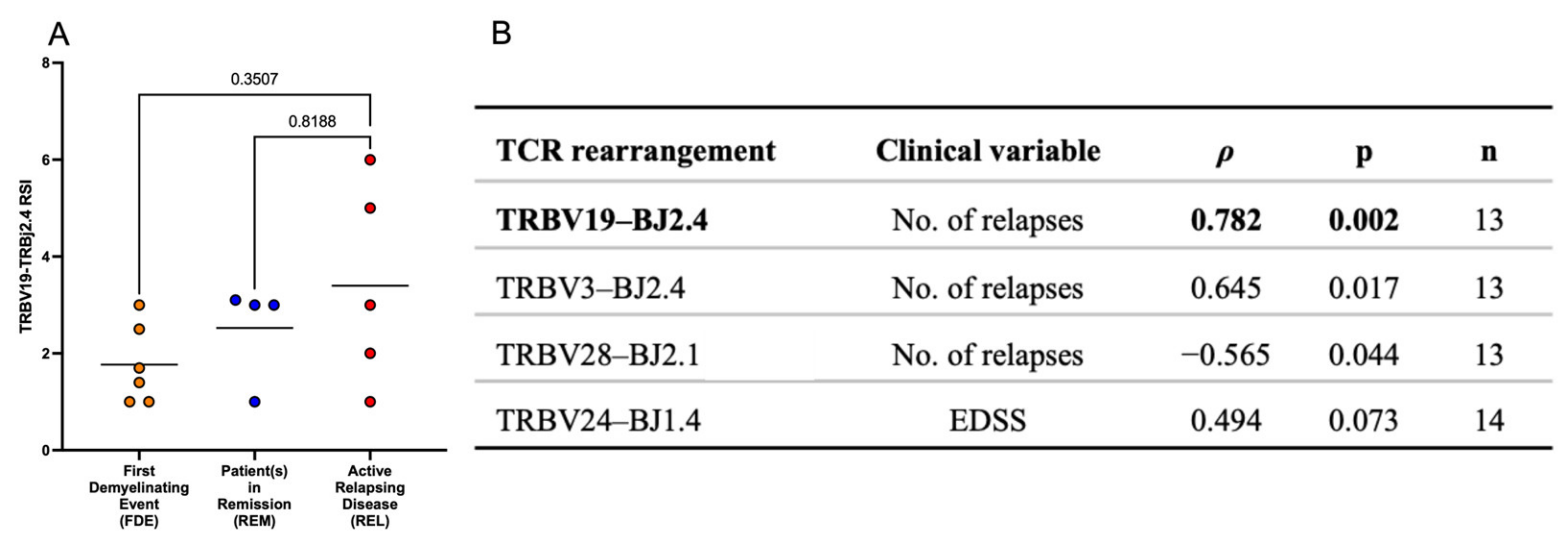
(**A**) TRBV19–BJ2.4 RSI values across disease groups in HLA-DRB1*15:01^+^ PwMS. Individual RSI values are shown for the first demyelinating event [FDE] (n = 6, orange), active relapsing disease [REL] (n = 4, blue) and remission [REM] (n = 5, red). Horizontal bars indicate group means ± SD. Spearman ρ = 0.782 (vs. number of relapses, *p* = 0.002, n = 13). RSI, Rate Stimulation Index; the Kruskal–Wallis omnibus test across the three groups was not significant (*p* = 0.27); *p*-values above brackets indicate pairwise comparisons of mean ranks, with false discovery rate controlled by the original Benjamini and Hochberg method (all non-significant). (**B**) Spearman rank correlations between MBP85–99-specific TCR rearrangement RSI values and clinical parameters (n = 13–15 HLA-DRB1*15:01^+^ subjects). Bold values indicate *p* < 0.01. TRBV28–BJ2.1 shows an inverse correlation consistent with its phase-restricted expression pattern. RSI, Rate Stimulation Index. Of the 15 subjects in the follow-up sub-cohort, relapse count data were available for 13; EDSS data were available for all 15. Accordingly, Spearman correlations involving relapse number were computed on n = 13, while those involving EDSS used n = 14–15 as indicated.

The strongest association observed was between the RSI of TRBV19-TRBJ2.4, the principal disease-associated public rearrangement identified in this study, and the number of clinical relapses (Spearman ρ = 0.782, *p* = 0.0016, n = 13). This finding is consistent with the hypothesis that clonal expansion of TRBV19-TRBJ2.4-bearing T cells reflects cumulative antigen-driven immune activation proportional to the inflammatory burden of the disease (Figure 5). A similar, albeit weaker, directional trend was observed for TRBV3-TRBJ2.4 (ρ = 0.645, *p* = 0.017, FDR q = 0.081). Conversely, the TRBV28-TRBJ2.1 rearrangement showed an inverse correlation with relapse number (ρ = −0.565, *p* = 0.044), potentially reflecting its more dynamic, phase-restricted expression pattern during acute inflammatory episodes rather than cumulative disease burden. The TRBV24-TRBJ1.4 rearrangement showed a trend toward positive correlation with EDSS (ρ = 0.494, *p* = 0.073), which did not reach statistical significance in this limited sample. No significant association was observed between TRBV2-TRBJ2.6, the health-associated rearrangement, and clinical severity parameters, consistent with its proposed role as a marker of non-pathogenic autoreactivity rather than active inflammatory disease. The total number of expanded rearrangements per patient positively correlated with the RSI of TRBV24-TRBJ1.5 (ρ = 0.718, *p* = 0.003) and showed a trend with TRBV19-TRBJ2.4 (ρ = 0.473, *p* = 0.075), suggesting that broader repertoire activation accompanies higher individual expression of disease-associated clones. These exploratory correlations, while limited by sample size and the cross-sectional design, support the biological coherence of the TCR signatures identified and reinforce the rationale for prospective validation in larger longitudinal cohorts.

## 3. Discussion

In this study, HLA-DRB1*15:01-positive persons with MS harbored a restricted set of shared (‘public’) TCRβ rearrangements specific for the immunodominant myelin epitope MBP85–99, enriched during active disease, reduced during remission, and qualitatively distinct from those observed in HLA-matched healthy donors, while a separate rearrangement was preferentially detected in healthy individuals and in persons with a first demyelinating event who did not convert to clinically definite MS.

Autoreactive T cells directed against myelin antigens have long been implicated in MS pathogenesis, but their clinical relevance has been difficult to establish because myelin-reactive cells are also present in healthy individuals [17,27,41,42,43]. Our data suggest that it is the clonotypic architecture, not mere antigen recognition, that tracks with MS: disease-associated public TCRs (active phases) versus a distinct (protective?) rearrangement characterizes the healthy response, helping distinguish pathogenic from ‘health-associated’ autoreactivity [26,28,44,45,46], although this observation is based on small subgroup numbers and requires prospective validation.

The broader MBP85–99-reactive repertoire observed at disease onset compared with established active MS may reflect dynamic clonal remodeling during disease evolution, whereby early inflammatory phases recruit a broad array of autoreactive clones, followed by selection and persistence of a more restricted disease-promoting subset during subsequent relapses, consistent with models of repertoire contraction in autoimmunity [26,47,48] and with evidence that HLA risk alleles shape TCR repertoire composition in MS [12]. The detection of disease-associated MBP85–99-reactive TCRs in CSF during active disease, but not remission, strengthens their biological plausibility: CSF offers a clinically accessible window into CNS immune activity, and compartment-specific immune enrichment has been confirmed in several single-cell studies [5,49]. The HLA-restriction of CSF rearrangement detection (markedly enriched in, though not entirely confined to, DRB1*15:01-positive persons; Section 2.3) further supports antigen-driven, MHC-restricted trafficking rather than non-specific inflammatory recruitment.

A related aspect concerns whether the immunodominant TRBV segments identified here are conserved across autoimmune diseases. TRBV20-1 over-representation has been reported both in antigen-specific CD4+ T cells reactive to citrullinated Tenascin-C [50] and in the broader synovial CD4+ T-cell repertoire of ACPA-positive, HLA-DRB1*04-carrying patients [51] and in muscle-infiltrating T cells in idiopathic inflammatory myopathies [52]. In each case, this reflects V-gene-level bias across a heterogeneous set of clones with different TRBJ genes and CDR3 sequences, rather than a shared rearrangement, and RA-associated TRBV20-1 clones have not been traced to a confirmed citrullinated-antigen specificity [51]. This differs from our findings, in which spectratyping combined with CDR3 sequencing identified specific, recurrent TRBV-TRBJ pairings (TRBV19–TRBJ2.4, TRBV24–TRBJ1.4) shared at the level of the paired V-J rearrangement and reinforced by convergent CDR3 motifs across unrelated HLA-DRB1*15:01-positive individuals with MS; none of our public/semi-public rearrangements used TRBV20-1.

Beyond the difference in HLA restriction driving selection of the autoreactive repertoire in each disease, this illustrates a difference in the granularity and in the strength of antigen-specific validation at which convergent selection has been demonstrated to date: TRBV19 (formerly Vβ17) usage by MBP-reactive T cells in HLA-DR2 individuals was already reported [53]; moreover a recurrent, DR2-restricted TRBV-TRBJ rearrangement was independently identified by spectratyping in newly diagnosed MS patients using a related V segment [54]; however a broader survey of all Vβ families found no consistent public, HLA-DR2-linked CDR3-length skewing, with MBP-reactive clones instead distributed across different, patient-specific Vβ families [55]. A subsequent CSF-restricted TRBV19 expansion was later reported, irrespective of DR2 status [56], indicating that V-J level restriction of the MBP-directed response has long-standing, if sparse and heterogeneous, experimental precedent. Within MS itself, the identity of dominant clonotypes is similarly not uniform across studies: high-throughput TCR sequencing of blood, CSF and CNS tissue has shown that public/shared clonotypes are frequently compartment- and cohort-specific [57], and unbiased TCR clustering approaches have implicated a range of TRBV segments without a single, consistently reproduced dominant clonotype [58].

Our identification of TRBV19–TRBJ2.4 and TRBV24–TRBJ1.4 as public, disease-associated, MBP85–99-specific rearrangements, therefore, adds a finer level of antigen-specific resolution to a broader literature in which the autoreactive TCR repertoire in MS, as in other autoimmune diseases, appears to be shaped jointly by HLA background and target epitope. Notably, TRBV19 usage per se is not restricted to MBP-reactive responses, having also been reported in CD8+ clonotypes of ambiguous or non-myelin specificity (including Epstein–Barr virus/influenza-associated clones) in the CSF of PwMS [59], underscoring that specificity in our data rests on the combined V-J pairing and recurrent CDR3 motif rather than V-segment usage alone.

The contraction of the circulating MBP85–99-reactive repertoire during IFN-β-induced remission suggests that antigen-specific TCR signatures may represent dynamic biomarkers of immunological disease activity, complementing rather than replacing established measures, including neurofilament light chain (NfL) and MRI activity [13,39,60,61]. Consistent with this, in the 15 subjects with both spectratyping data and ~10-year clinical follow-up, the RSI of TRBV19–TRBJ2.4 correlated positively with cumulative clinical relapses (Spearman ρ = 0.782, *p* = 0.002; Figure 5, Table 1), independently of disease group membership, indicating that TRBV19–TRBJ2.4 expansion reflects cumulative antigen-driven immune activation rather than merely acute disease status. A directionally similar, albeit weaker, association was observed for TRBV3–TRBJ2.4 (ρ = 0.645, *p* = 0.017), while TRBV28–TRBJ2.1 showed an inverse relationship with relapse burden (ρ = −0.565, *p* = 0.044), consistent with a more dynamic, phase-restricted expression pattern. These exploratory findings support the biological coherence of the identified public TCR signatures and warrant prospective evaluation as candidate predictors of long-term inflammatory disease activity [40,60].

The recurrent re-emergence of disease-associated rearrangements at relapse after apparent quiescence during remission raises the question of what sustains the capacity of antigen-experienced T cells to repeatedly re-enter the CNS. Toll-like receptor (TLR) signaling and CD44 expression on memory T cells may be relevant in this context: network-based transcriptomic analysis of MS gene expression datasets has identified CD44 as the highest-connectivity hub gene in the MS protein–protein interaction network, with significant upregulation in MS gray matter tissue [62], and CD44 spliced variants regulate the migratory properties of CNS-infiltrating immune cells in MS and EAE [29,63]. Given that the blood–brain barrier displays regional transcriptional specializations that may differentially regulate leukocyte access to CNS compartments [64], innate immune triggers such as infections, well-established precipitants of clinical relapses [65], could sustain or re-amplify CNS-directed T-cell responses through TLR-mediated reactivation of CD44hi memory clones without requiring renewed antigen presentation. Whether the public MBP85–99-reactive rearrangements identified in this study preferentially belong to CD44hi effector-memory subsets and whether their reactivation is facilitated by TLR co-stimulation represents a testable hypothesis for future mechanistic studies.

The main limitations of this study include its modest cohort size, partly attributable to restricting the analysis to HLA-DRB1*15:01-positive persons (~30% of the MS population, the highest-risk genotype), the use of McDonald criteria applicable at the time of patient classification [66,67] and the use of spectratyping/immunoscope technology rather than high-throughput immune repertoire sequencing. Spectratyping was performed on total peripheral blood mononuclear cell cultures rather than on sorted CD4+ T cells; a contribution from CD8+ or bystander-activated populations cannot be formally excluded, and activation status was not directly confirmed by surface markers (e.g., CD25, CD69, OX40/CD137) as in AIM-based assays. Conversion to clinically definite MS among FDE participants was ascertained within a standardized 6-month follow-up window applied uniformly to all eight participants; while this ensured equal observation time when comparing carriers and non-carriers of the TRBV2–TRBJ2.6 rearrangement, later conversion beyond this window cannot be excluded, and the small number of participants (n = 8) limits statistical power for this specific comparison. The reduced antigen-specific expansion observed in healthy controls compared with persons with MS may reflect either a lower frequency of genuinely antigen-specific responses or reduced bystander activation associated with lower baseline systemic inflammation. Although none of the pairwise RSI comparisons remain statistically significant after FDR correction applied across the 20 candidate rearrangements screened (Section 4.11; Supplementary Table S6), their classification as public rearrangements rests on their pre-defined sharing pattern across unrelated subjects (Section 4.7) and on converging independent evidence (recurrent CDR3 motifs, CSF enrichment, and longitudinal correlation with relapse burden). Notably, the correlation of TRBV19–TRBJ2.4 with cumulative relapses, of TRBV24–TRBJ1.5 with overall repertoire breadth, and all three CSF enrichment comparisons remained statistically significant after Benjamini–Hochberg correction (q < 0.05; Supplementary Table S6). Among the 20 screened rearrangements, TRBV3–TRBJ2.4 showed the next most consistent signal across both cross-sectional (Kruskal–Wallis q = 0.067) and longitudinal (Spearman q = 0.081) analyses and represents an additional candidate warranting future validation.

Nevertheless, these limitations also define a clear framework for future validation. The focused set of candidate rearrangements identified here is directly amenable to investigation in larger independent cohorts using bulk TCR sequencing, single-cell TCR/RNA sequencing, and antigen-reactive T-cell enrichment approaches [68,69], including longitudinal sampling across relapses, remission and treatment initiation. Spectratyping remains a validated approach for detecting antigen-driven clonal expansions and has been applied successfully in multiple autoimmune contexts [22,24,30,31], enabling sensitive identification of TRBV-TRBJ combinations expanded against a polyclonal background, based on perturbation of CDR3 length distributions, a parameter lost in bulk TCR sequencing. It nonetheless offers substantially lower resolution than contemporary high-throughput TCR sequencing, which achieves single-nucleotide, single-clonotype resolution and reports absolute clonal frequencies, while MHC class II tetramer/dextramer-based approaches remain the gold standard for unambiguous identification of antigen-specific CD4+ T cells [70,71,72,73]. A key strength of the present approach, however, is that it yielded a small, biologically prioritized list of candidate rearrangements (n = 3 public rearrangements) with defined CDR3 sequences (Tables S1 and S2), directly amenable to validation using bulk TCRβ sequencing in independent cohorts, antigen-reactive T-cell enrichment with pMHC multimers or MIRA-based platforms, and paired scRNA-seq/TCR-seq for functional annotation [68,69]. In conclusion, our findings suggest that MS is associated not merely with the presence of myelin-reactive T cells but with distinct public antigen-specific TCR signatures associated with inflammatory disease activity. These results support further validation of MBP85–99-reactive public rearrangements as candidate biomarkers and provide a rationale for integrating antigen-specific TCR profiling into next-generation biomarker studies in MS.

## 4. Materials and Methods

### 4.1. Study Population

Eighty-five consecutive subjects with demyelinating disease suggestive of multiple sclerosis (MS) were recruited at the Multiple Sclerosis Centers of Fondazione Policlinico Universitario A. Gemelli IRCCS (Rome, Italy). After HLA genotyping, 30 subjects carrying at least one HLA-DRB1*15:01 allele were included in the present study. The final study cohort comprised 8 patients at first demyelinating event (FDE), 11 patients with active relapsing disease (REL) and 11 patients with stable disease in remission (REM). Of these, 15 subjects for whom long-term clinical follow-up records (approximately 10 years) were available are described separately in Table 1 and Section 2.5.

Diagnosis of the first demyelinating event and MS was established according to the McDonald diagnostic criteria applicable at the time of patient classification [66,67]. Persons classified as REL had evidence of recent clinical relapse (within 30 days prior to sampling) and/or gadolinium-enhancing lesions on MRI performed within 30 days of sampling and had received no corticosteroid treatment in the 30 days preceding blood draw. Persons classified as REM had no clinical relapse in the 6 months preceding sampling and no new or enlarging T2 lesions or gadolinium-enhancing lesions on the most recent MRI available at the time of enrolment.

At sampling, patients had not received immunosuppressive therapy within the previous 24 months and had discontinued immunomodulatory treatment for at least 6 months. As controls, 13 healthy relatives of MS patients were screened, and 7 HLA-DRB1*15:01-positive subjects were included as HLA-matched healthy donors. Demographic and clinical characteristics of the TCR study cohort are summarized in Table 2. Follow-up data for the 15 subjects with complete clinical records are provided in Table 1.

### 4.2. Longitudinal Follow-Up Sub-Cohort

Of the 30 subjects included in the TCR spectratyping study, 15 individuals (6 with a first demyelinating event, 4 with active relapsing disease, 5 PwMS in remission at the time of sampling) had complete clinical follow-up records available in the institutional database at approximately 10 years after initial enrollment. For these subjects, EDSS, accumulated relapse count, current disease-modifying therapy (DMT), and MRI follow-up status were retrieved and integrated with individual-level RSI data for the cross-sectional correlation analyses described in Section 2.5. Demographic and follow-up characteristics of this sub-cohort are summarized in Table 1. Subjects without available follow-up records were either enrolled after April 2016 or did not attend the institutional MS clinic during the follow-up observation window.

### 4.3. Ethics Statement

All participants provided written informed consent before enrollment. Peripheral blood and, when clinically indicated, cerebrospinal fluid (CSF) samples were collected after consent and anonymized using coded identifiers. This study was conducted according to the Declaration of Helsinki and approved by the Institutional Review Board (or Ethics Committee) of Università Cattolica del Sacro Cuore of Roma (protocol code 0023390/17, 11 May 2017).

### 4.4. Genotyping of MS Patients

Genomic DNA was extracted from peripheral blood using QIAamp DNA Mini Kits (Qiagen, Hilden, Germany), according to the manufacturer’s instructions. HLA-DRB1 typing was performed by polymerase chain reaction (PCR) using sequence-specific oligonucleotide probes with the Inno-LiPA HLA-DRB1 Amp Plus kit (FUJIREBIO Inc., Tokyo, Japan). Subjects were included in the final cohort only when genotyping demonstrated the presence of at least one HLA-DRB1*15:01 allele, the strongest genetic susceptibility factor for MS identified across populations [8,9].

### 4.5. Isolation and Culture of Peripheral Blood Mononuclear Cells

Peripheral blood mononuclear cells (PBMCs) were isolated from EDTA-anticoagulated blood by density-gradient centrifugation (Cedarlane, Burlington, ON, Canada). PBMCs were cultured in RPMI 1640 medium (Thermo Fisher, Waltham, MA, USA) supplemented with L-glutamine, antibiotics, and human AB serum (Thermo Fisher, Waltham, MA, USA). Cells were plated at 5 × 10^6^ cells/well and stimulated for 72 h with synthetic peptides derived from myelin basic protein (MBP): MBP85–99, an immunodominant epitope presented by HLA-DRB1*15:01; and MBP111–129, used as a comparator peptide. Parallel unstimulated cultures were used as controls. MBP85–99 is one of the most extensively studied myelin epitopes in HLA-DRB1*15:01-positive MS and can activate autoreactive CD4+ T cells [16,17]. After culture, viable cells were recovered and processed for TCR repertoire analysis.

### 4.6. T-Cell Receptor Repertoire Analysis

Antigen-specific T-cell receptor (TCR) β-chain repertoires were analyzed using immunoscope/spectratyping, a reverse transcription-PCR (RT-PCR)-based method that detects expansions of specific TRBV-TRBJ rearrangements by CDR3 length distribution analysis [22,31,74,75].

Total RNA was extracted from cultured or freshly isolated cells using RNeasy Mini Kits (Qiagen, Hilden, Germany), reverse-transcribed into cDNA using Omniscript (Qiagen, Hilden, Germany), and amplified using combinations of TRBV family-specific forward primers and common TRBC reverse primers. Fluorescent run-off reactions with TRBJ-specific primers were then performed, and products were analyzed by capillary electrophoresis on an ABI 3130 Genetic Analyzer (Thermo Fisher, Waltham, MA, USA).

Each peak corresponded to a CDR3 fragment length differing by three nucleotides. Antigen-driven clonal expansion was identified by perturbation of the Gaussian distribution observed in unstimulated control cultures. Results were expressed as Rate Stimulation Index (RSI), calculated as RSI = normalized peak area in antigen-stimulated culture/normalized peak area in unstimulated culture. A rearrangement was considered expanded when RSI ≥ 2, according to previously validated criteria [31]. Candidate rearrangements shown in Figure 1A,B were those meeting this RSI ≥ 2 enrichment criterion in the antigen-stimulated culture relative to the paired unstimulated control, rather than rearrangements exclusively present only in the stimulated condition.

### 4.7. Definition of Public and Private TCR Rearrangements

Shared expansions detected in multiple HLA-matched individuals in response to the same antigen were defined as public rearrangements, whereas rearrangements detected only in individual subjects were considered private rearrangements. Rearrangements recurrently detected in a substantial proportion of subjects, but not universally shared, were considered semi-public rearrangements [75]. This terminology is consistent with current concepts of convergent adaptive immune responses and recurrent TCR generation across individuals [76,77,78].

### 4.8. CDR3 Sequencing of Selected Rearrangements

Selected public or semi-public rearrangements were further characterized by nested PCR amplification of TRBV-TRBJ products, cloning into plasmid vectors, and Sanger sequencing. Nucleotide sequences were translated into amino acid sequences and analyzed to identify recurrent complementarity-determining region 3 (CDR3) motifs suggestive of antigen-driven convergent selection [22,24].

### 4.9. Cerebrospinal Fluid Samples

CSF samples obtained for clinical diagnostic purposes were available from a subset of patients. Mononuclear cells recovered from CSF were directly analyzed for the presence of selected disease-associated TRBV-TRBJ rearrangements without prior in vitro stimulation. CSF immune profiling is increasingly recognized as a valuable window into compartmentalized CNS inflammation in MS [5,65,79,80].

### 4.10. Longitudinal Analysis During Interferon Beta Treatment

A subset of patients underwent serial peripheral blood sampling before and during treatment with interferon beta-1a. Samples obtained at baseline and follow-up visits were analyzed to assess longitudinal changes in the MBP85–99-reactive TCR repertoire in relation to clinical disease activity. Interferon beta is an established therapy for relapsing MS and exerts immunomodulatory effects on T-cell activation, cytokine signaling, and leukocyte trafficking [38,39,81].

### 4.11. Statistical Analysis

Statistical analyses were performed using GraphPad Prism 11 (San Diego, CA, USA). Continuous variables are reported as mean ± standard deviation (SD) or median (interquartile range), as appropriate. Between-group comparisons of individual-level, single-rearrangement RSI values (Figure 2 and Figure 5A, Supplementary Table S6) were performed using Mann–Whitney U or Kruskal–Wallis tests, as appropriate given the skewed distribution of RSI values in small subgroups. For the aggregate TCR-usage-frequency comparisons across the four disease-status groups (Figure 1B,D), an ordinary one-way ANOVA was used after confirming that homogeneity of variance (Brown–Forsythe test) and normality (Bartlett’s test) assumptions were not violated. Categorical variables were analyzed using Fisher’s exact test or chi-square test, according to sample size assumptions. A two-sided *p* value < 0.05 was considered statistically significant. A complete summary of all statistical tests performed, including test statistics, sample sizes, and *p*-values, is provided in Supplementary Table S6. No formal correction for multiple comparisons (e.g., Bonferroni or false discovery rate) was applied, as this study was designed as hypothesis-generating rather than as a pre-specified confirmatory analysis; the resulting risk of false-positive findings is discussed as a limitation below. For Figure 1B,D, pairwise comparisons following the omnibus ANOVA were corrected for multiple testing using the two-stage step-up method of Benjamini, Krieger and Yekutieli, as indicated in the figure legend. For Figure 5A, pairwise comparisons of mean ranks following the Kruskal–Wallis test were corrected using the original false discovery rate (FDR) method of Benjamini and Hochberg, as indicated in the figure legend. Outside of these two figures, no formal correction for multiple comparisons was applied to the exploratory, hypothesis-generating screen of the 20 candidate rearrangements (Figure 2, Supplementary Table S6); the resulting risk of false-positive findings, and a sensitivity analysis applying Benjamini–Hochberg FDR correction across this full screen, are discussed in the Limitations paragraph below.

## 5. Conclusions

In conclusion, this study suggests that HLA-DRB1*15:01-restricted MBP85–99-specific public TCR signatures are associated with inflammatory disease activity in MS. A focused set of candidate rearrangements was identified that distinguishes active disease from remission and from the autoreactive repertoire present in healthy individuals. By linking antigen-specific TCR architecture to clinical disease activity, our findings establish a rationale for integrating immune repertoire profiling into next-generation biomarker strategies and provide a framework for investigating pathogenic T-cell responses in MS at clonal resolution. Preliminary longitudinal data further suggest that the magnitude of TRBV19–TRBJ2.4 clonal expansion correlates with cumulative inflammatory burden over a ~10-year period, providing an initial proof-of-concept for the prospective use of antigen-specific TCR profiling as a dynamic biomarker of long-term disease activity.

## Figures and Tables

**Table 1:** Clinical characteristics and ~10-year follow-up data for the 15 HLA-DRB1*15:01^+^ subjects with available TCR spectratyping data (sampled before April 2016; see Section 2.5, Figure 5). Diagnosis refers to clinical status at the time of sampling. Data are mean ± SD unless otherwise stated. † EDSS at follow-up available for 5/6 FDE, 2/4 REL, 3/5 REM. DMT, disease-modifying therapy; GA, glatiramer acetate; IFN-β, interferon-beta; MRI, magnetic resonance imaging; S1P, sphingosine-1-phosphate receptor modulator.

Variable	First Demyelinating Event (n = 6)	Active Relapsing Disease (n = 4)	Remission (n = 5)
Age at sampling, years	30.2 ± 4.2	28.0 ± 6.0	35.8 ± 12.6
Female sex, n (%)	5 (83%)	3 (75%)	3 (60%)
EDSS at sampling	1.20 ± 0.84	1.67 ± 0.58	0.80 ± 0.45
EDSS at ~10-year follow-up †	0.80 ± 0.76	1.75 ± 1.06	1.50 ± 0.87
Relapses accumulated at follow-up	1.0 ± 0.0	1.7 ± 0.6	1.5 ± 0.6
Oligoclonal bands, n (%)	4 (80%)	2 (100%)	2 (100%)
Current DMT, n (%)	3 (50%)	2 (50%)	2 (40%)
Platform (IFN-β/GA)	1 (33%)	—	—
Dimethyl fumarate	2 (67%)	—	1 (50%)
Anti-CD20	—	1 (50%)	—
Alemtuzumab	—	1 (50%)	—
S1P inhibitors	—	—	1 (50%)
MRI follow-up available, n (%)	5 (83%)	2 (50%)	3 (60%)
Stability	5 (100%)	1 (50%)	3 (100%)
Active lesions (Gd+)	—	1 (50%)	—

**Table 2:** Demographic and clinical characteristics of the TCR study cohort (n = 30 HLA-DRB1*15:01^+^ subjects sampled before April 2016, plus 7 HLA-matched healthy donors).

HLA-DRB1*15:01+ Patients	First Demyelinating Event (FDE)	Active Relapsing Disease (REL)	Remission (REM)	Healthy Donors (HDs)
Number (% enrolled)	8 (9.4)	11 (12.9)	11 (12.9)	7
Age (years)	28.7 ± 5.7	31 ± 7.6	34.3 ± 10.8	35.2 ± 6.3
Sex, female n (%)	6 (75%)	7 (63.6%)	6 (54.5%)	5 (71.4%)
Disease duration (months)	2.4 ± 2.1	14.5 ± 5.6	105.8 ± 152.3	N/A
EDSS at sampling	1.25 ± 0.76	1.72 ± 0.98	0.78 ± 0.57	N/A
GD+ lesions at sampling (%)	87.5	72.7	27.2	N/A
Oligoclonal bands (%)	87.5	67.7	54.5	N/A

## Data Availability

The original contributions presented in this study are included in the article. Further inquiries can be directed to the corresponding author.

## Supplementary Materials

The following supporting information can be downloaded at: https://www.mdpi.com/article/10.3390/ijms27157034/s1.

## Abbreviations

The following abbreviations are used in this manuscript:

BBB	blood–brain barrier
CDR3	complementarity-determining region 3
FDE	first demyelinating episode
CNS	central nervous system
CSF	cerebrospinal fluid
DMT	disease-modifying therapy
EAE	experimental autoimmune encephalomyelitis
EDSS	Expanded Disability Status Scale
GA	glatiramer acetate
GD+	gadolinium-enhancing
HD	healthy donors
HLA	human leukocyte antigen
IFN-β	interferon-beta
IFN-γ	interferon-gamma
MBP	myelin basic protein
MHC	major histocompatibility complex
MRI	magnetic resonance imaging
MS	multiple sclerosis
NfL	neurofilament light chain
PBMC	peripheral blood mononuclear cells
PLP	proteolipid protein
RA	rheumatoid arthritis
REL	active relapsing disease
REM	stable disease in remission
RSI	Rate Stimulation Index
S1P	sphingosine-1-phosphate
SJL/J	Swiss Jim Lambert mice
TCR	T-cell receptor
TLR	Toll-like receptor
TRBC	T-cell receptor beta constant region
TRBJ	T-cell receptor beta joining gene segment
TRBV	T-cell receptor beta variable gene segment
